# The association of mood disorders and mild cognitive impairment with language performance in older adults: insights from a community healthcare setting

**DOI:** 10.3389/frdem.2026.1739429

**Published:** 2026-06-01

**Authors:** Nomiki Karpathiou, Angeliki Tsapanou, Areti Efthymiou, Evdoxia Kotsiou, Xanthi Arampatzi, Panagiota Zoi, Faidra Kalligerou, Paraskevi Sakka

**Affiliations:** 1 Department of Speech and Language Therapy, University of the Peloponnese, Kalamata, Greece; 2 Athens Alzheimer’s Association, Athens, Greece; 3 Department of Speech and Language Therapy, University of Patras, Patras, Greece

**Keywords:** cognitive decline, dementia, depression, language impairment, MCI, naming, phonemic fluency, verbal fluency

## Abstract

**Background:**

Mood disorders are frequent in memory clinic populations, but their association with language difficulties in the context of cognitive decline is not well understood. The study aimed to examine the association of cognitive decline and mood disorders with two aspects of language performance: verbal fluency and confrontation naming.

**Methods:**

A total of 183 participants were included in the study. Specifically, 92 individuals with a prior mood disorder diagnosis were categorized by cognitive status into two groups: 48 with mild cognitive impairment (MCI) and 44 neurotypical adults. A random sample of 91 individuals (44 with MCI and 47 without MCI) formed the control group. Two regression models were tested for each variable: verbal fluency and naming. Model A included cognitive status, mood disorder diagnosis, and demographic variables, while Model B further incorporated the Geriatric Depression Scale (GDS) as a measure of depressive symptom severity. Two additional regression models, identical in predictor structure to Model A, were estimated for phonemic and semantic fluency.

**Results:**

The results indicate that cognitive decline was the strongest predictor of both verbal fluency and naming, with demographic factors influencing verbal fluency performance. Older age and lower education were associated with poorer performance. In separate models, cognitive status predicted both phonemic and semantic fluency, whereas mood disorder diagnosis was associated with phonemic but not category fluency. Depression severity did not independently predict either outcome.

**Discussion:**

These findings highlight the central role of cognitive status in driving language performance, including both naming and verbal fluency. In contrast, mood disorder diagnosis was selectively associated with phonemic fluency, which relies more on executive control. Current depressive symptom severity did not add explanatory value beyond diagnostic and demographic variables. Assessing these tasks separately may help clinicians distinguish mood-related cognitive difficulties from early cognitive decline.

## 1 Introduction

Population aging has emerged as one of the most pressing global challenges. According to the World Health Organization (2025), the number of individuals aged 60 years and above will reach 1.4 billion by 2030, while Eurostat (2025) projects that in Europe one in four citizens will be older than 65. This global trend is also evident in Greece, which has one of the highest proportions of older adults in Europe, with nearly 24% of its population aged 65 and over in 2024 (Eurostat, 2025). These demographic changes are leading to a growing prevalence of cognitive and mental health disorders among older adults.

Normal cognitive aging is marked by diminished executive functions such as inhibition and set-shifting, slowed processing speed and reduced working memory capacity (Mosti et al., 2019). While vocabulary and comprehension are largely maintained in older age, difficulties often arise in phonological retrieval (e.g., tip-of-the-tongue states, confrontation naming, and verbal fluency). These changes are primarily linked to decline in auditory attention and verbal memory rather than degradation of the lexical-semantic system itself. In connected speech, the rate may slow modestly, but narrative structure, coherence, and grammar remain intact (Peelle, 2019). Distinguishing between normal cognitive aging and pathological decline can be challenging without neuropsychological assessment that incorporates normative data.

Against this background, Mild Cognitive Impairment (MCI) is characterized by cognitive deficits that do not compromise daily functioning. According to the fifth edition of the Diagnostic and Statistical Manual of Mental Disorders (DSM-5) (APA, 2013), MCI involves modest decline in one or more cognitive domains, such as memory, attention, language, or executive function, based on concerns expressed by the individual, an informant, or a clinician. This decline should be confirmed through neuropsychological testing. MCI is clinically significant, as it may represent a prodromal stage of Alzheimer’s disease or other dementias. However, conversion rates may vary (McGrattan et al., 2022; Salemme et al., 2025). A recent systematic review of 89 studies with a mean follow-up of 5.2 years (Salemme et al., 2025) found that the risk of conversion to dementia was 41.5% (95% CI: 38.3–44.7%) in clinical cohorts and 27.0% (95% CI: 22.0–32.0%) in community-based samples. The corresponding annual conversion rates to dementia were 10.9% (95% CI: 9.7–12.1%) in clinical populations and 5.9% (95% CI: 4.9–6.9%) in community samples.

MCI is a heterogeneous syndrome, and language profiles vary depending on MCI subtype (amnestic and non-amnestic; single- and multi-domain), underlying pathology (e.g., Alzheimer’s disease, vascular cognitive impairment, mixed), disease stage, and task demands. MCI is associated with language impairments at both the micro- and macrolinguistic levels. Discourse analysis in individuals with MCI reveals reduced coherence (the ability to remain on topic), impaired cohesion, reflected in the less frequent or less effective use of cohesive markers such as pronouns and conjunctions, along with lower propositional density, defined as the amount of meaningful content conveyed (Kim et al., 2019). Fluency is disrupted by increased hesitations, fillers, and pauses (Badal et al., 2024). At the lexical level, MCI is associated with reduced richness of vocabulary, increased word-finding difficulties, and greater reliance on pronouns, all of which compromise discourse informativeness (Badal et al., 2024). Naming deficits and tip-of-the-tongue errors also emerge early in the course of MCI, with multiple studies documenting declines in confrontation naming tasks compared to healthy aging (Karstens et al., 2024.; Liampas et al., 2023). Overall, individuals with MCI exhibit discourse impairments, indicating that language abilities may be vulnerable in the early stages of cognitive decline.

Mood disorders are frequently accompanied by cognitive impairments. The domains most consistently affected include attention, processing speed, working memory, verbal learning, and episodic memory. Importantly, these deficits are observed across both major depressive disorder and bipolar disorder (Galimberti et al., 2020). In turn, affective disorders can influence language by impairing attention, working memory, and executive functions, which are all critical for effective communication. Research shows that mood disorders leave their identifiable “linguistic imprints” across multiple domains of language, including lexical, semantic, syntactic, and pragmatic components (Koops et al., 2023; Trifu et al., 2024).

Moreover, evidence suggests that greater depression severity and longer duration of untreated illness are both associated with poorer cognitive performance (Galimberti et al., 2020). Supporting this view, a population-based longitudinal study conducted in Korea concluded that both the intensity and duration of depressive symptoms are significantly associated with accelerated cognitive decline in older adults (Lu et al., 2025). Individuals with major depression showed greater and more rapid decline in global cognition, language, and attention/calculation compared to those with mild depression. The findings underscore the importance of early detection and treatment of depression in mitigating long-term cognitive decline.

Verbal fluency tasks are widely used to evaluate language, executive control, and semantic memory in both mood disorders and neurocognitive impairment. Cognitive architecture studies indicate that phonemic (letter) and semantic (category) fluency engage overlapping but partially distinct processes. Specifically, phonemic fluency places greater demands on strategic search and executive control, whereas semantic fluency is more strongly influenced by memory processes. This complex organization reflects interactions among lexico-semantic storage, strategic search, and attentional control (Dorchies et al., 2024). In major depressive disorder, systematic reviews indicate that verbal fluency impairments are most consistently observed in the semantic domain, whereas findings for phonemic fluency are more variable, suggesting that mood disorders may preferentially affect lexical-semantic retrieval and related cognitive resources (Kriesche et al., 2023). In contrast, in aging and cognitive decline, evidence from a recent meta-analysis shows marked reductions in both phonemic and semantic fluency in MCI and Alzheimer’s disease, with semantic fluency often showing stronger impairment due to deterioration of semantic networks, whereas phonemic performance can be comparatively less impaired early in the disease process (Olmos-Villaseñor et al., 2023). Thus, separating phonemic and semantic fluency can clarify whether observed fluency deficits reflect executive search limitations, semantic memory breakdown, or both in populations with mood disorders and cognitive impairment.

Although both MCI and mood disorders are common in older adults, relatively few studies have investigated their association with language functioning. The present study was designed to address this gap by examining how these conditions influence two key aspects of language performance: verbal fluency and naming. The primary aim of the study was to evaluate the association of mood disorders and cognitive decline with language performance. In addition, the study explored the moderating role of demographic variables (age, education and sex), as well as severity of depressive symptoms, given their known associations with cognitive and linguistic outcomes.

## 2 Materials and methods

### 2.1 Study design

The study employed a retrospective design based on a review of medical records from individuals attending the memory clinic of Alzheimer Athens’ Daycare Center in Pagrati.

### 2.2 Procedures

MCI diagnosis was established in expert consensus meetings held at the memory clinic. The panel consisted of experienced neurologists and clinical neuropsychologists specialized in cognitive disorders. The consensus process integrated findings from a neurological examination, neuropsychological testing covering auditory and visual memory, attention, processing speed, executive functions, language, and visuospatial abilities, and functional assessment using the Instrumental Activities of Daily Living scale (IADL) (Lawton and Brody, 1969; Theotoka et al., 2007) and the Clinical Dementia Rating scale (CDR) (Morris, 1993). Neuroimaging (MRI/CT) and routine laboratory investigations were reviewed by neurologists to support diagnosis and exclude alternative causes. Diagnoses were established according to DSM-5 criteria for major or mild neurocognitive disorder, with etiological classification based on established disease-specific criteria (e.g., the National Institute on Aging–Alzheimer’s Association [NIA-AA] clinical criteria for Alzheimer’s disease; Albert et al., 2011).

Mood disorder diagnosis was determined based on participant self-report of a current diagnosis, previously established by a healthcare professional. For participants reporting a mood disorder, current medication use (e.g., antidepressants, anxiolytics, mood stabilizers, antipsychotics) was documented at the time of assessment. Past medication history was not systematically collected and no confirmation from treating psychiatrists was obtained. Episode duration and number of prior episodes were not systematically documented. The examining neurologists assigned diagnoses and corresponding codes using the International Statistical Classification of Diseases and Related Health Problems, 10th Revision (ICD-10) (World Health Organization, 2019), in accordance with clinical criteria.

The study sample was drawn from individuals evaluated at the memory clinic during a nine-month data collection period. Individuals with a diagnosis of dementia, other neurological diagnoses, or neoplastic conditions were excluded from the sampling pool. We also excluded cases with missing data in key study variables, as well as cases with inconclusive diagnostic classification.

Among eligible attendees, 92 individuals reported a previously established diagnosis of a mood disorder and were included in the study. These participants were classified according to cognitive status into two groups: mild cognitive impairment (MCI; *n* = 48) and neurotypical cognition (*n* = 44).

To form a control group without a history of mood disorder, a sampling pool was constructed from the remaining eligible attendees without a reported mood disorder. Within this pool, 763 individuals were classified as MCI and 1,136 as neurotypical. Participants were then randomly selected separately within each cognitive category using the RAND() function in Microsoft Excel to obtain approximately equal numbers (MCI: *n* = 44; neurotypical: *n* = 47), resulting in a control group of 91 individuals.

The final sample comprised four subgroups: (a) MCI with mood disorder (*n* = 48), (b) neurotypical with mood disorder (*n* = 44), (c) MCI without mood disorder (*n* = 44), and (d) neurotypical without mood disorder (*n* = 47). This design enabled cross-comparisons to explore how mood and cognitive factors may influence language performance.

### 2.3 Participants

In total, 183 participants were included in the present analysis. Inclusion criteria required the availability of complete neurological and neuropsychological assessments and the absence of a dementia diagnosis. The sample was predominantly female (84.7%), with a mean age of 70.79 years (SD = 6.03) and a mean of 12.97 years of formal education (SD = 3.87).

Participants with a mood disorder diagnosis were classified by the examining neurologists according to clinical criteria under the corresponding ICD-10 code. The majority (*n* = 88) fell into the ICD-10 category F34 for persistent mood disorders, which includes mood disorders that are chronic but not severe enough to qualify as major depressive disorder or bipolar disorder. Four individuals were classified under the code F38 used for mood disorders that do not meet the criteria for major depression or bipolar disorder and are not classified under other codes. These disorders may have atypical features, incomplete profiles, or represent mixed patterns.

Anxiety symptoms were also documented in 15/92 participants (16.3%) based on clinical interview and self-report. In these cases, anxiety was noted as a secondary clinical feature and was not assessed in detail. Given its low prevalence and limited available information, anxiety was not analysed as a separate diagnostic category in the present study.

### 2.4 Measures

The outcome measures were part of a comprehensive neuropsychological battery designed to assess cognition, language performance, and mood. The primary outcomes were verbal fluency and naming tasks extracted from the Greek version of the Addenbrooke’s Cognitive Examination–Revised (ACE-R) (Konstantinopoulou et al., 2011; Mioshi et al., 2006), which served as the main indicators of language performance. Verbal fluency was assessed using two distinct tasks: phonemic (letter) fluency and semantic (category) fluency. In the phonemic fluency task, participants had to generate as many words as possible beginning with the letter ‘*p*’ within one minute. In the semantic fluency task, they were asked to generate as many animals as possible within the same time limit. Phonemic and semantic fluency were analyzed both separately and as a composite verbal fluency measure, with the latter reflecting overall lexical retrieval and executive processes across different domains of word generation. The naming task assesses confrontation naming by requiring participants to identify pictured objects. It provides an index of semantic knowledge and lexical access.

In addition, the Greek short form of the Geriatric Depression Scale (GDS) (Fountoulakis et al., 1999; Sheikh and Yesavage, 1986) was included as a covariate to account for the severity of depressive symptoms in the analyses. For this scale, lower scores reflect lower severity.

### 2.5 Statistical analysis

Descriptive statistics were computed for all variables. Group comparisons between participants with and without a mood disorder were performed using independent samples t-tests for age and education and chi-square tests for sex.

To examine the association of mood disorders and cognitive impairment with language performance, analyses were conducted using ordinary least squares (OLS) regression for verbal fluency and naming. Two models were specified *a priori* for each outcome. Model A included cognitive status (neurotypical and MCI) and presence or absence of a mood disorder as primary predictors, and demographic variables, i.e., age, education, and sex, as covariates. Cognitive status and mood disorder diagnosis were entered simultaneously to estimate the association of each predictor with language performance while adjusting for the other and for demographic covariates, consistent with the study aim of examining the independent contributions of cognition and mood to language measures. Model B further included GDS. Mood disorder diagnosis and GDS scores capture related but distinct constructs: ICD-10 diagnosis reflects a categorical clinical classification, whereas the GDS provides a standardized measure of current depressive symptom severity at assessment. GDS was therefore included to test whether depressive symptom severity accounted for additional variance in language performance beyond diagnostic status.

Verbal fluency was also analysed by separating phonemic and semantic components. Separate regression models were fitted for phonemic and semantic fluency using the same Model A predictors and covariates, as evidence suggests that these tasks rely on partially distinct cognitive mechanisms.

Model assumptions were examined prior to interpretation. Residual diagnostics indicated heteroskedasticity for both fluency and naming, and deviations from normality for naming. As the normality assumption primarily affects inference rather than coefficient estimates in large samples, HC3 robust standard errors were calculated to obtain valid confidence intervals and *p*-values under heteroskedasticity and non-normality (Long and Ervin, 2000).

Model fit was evaluated using *R*^2^, adjusted *R*^2^, and Akaike Information Criterion (AIC). The contribution of depressive symptoms severity was assessed using a robust Wald test of its regression coefficient in Model B, and the difference in AIC (ΔAIC) between Model B and Model A. An improvement in model fit was inferred only if depressive symptom severity reached statistical significance and resulted in a reduction in AIC of 2 points or greater.

Effect sizes were computed alongside significance tests and Cohen’s d with 95% confidence intervals were calculated to quantify the magnitude of group differences in fluency and naming.

All analyses were conducted in Python (statsmodels v0.14) using ChatGPT-5 and cross-checked against SPSS (v29). To address assumption violations in SPSS, regression coefficients were estimated using bootstrapped standard errors (1,000 resamples, bias-corrected and accelerated [BCa] 95% confidence intervals).

### 2.6 Ethical considerations

The study was approved by the Ethics Committee of Athens Alzheimer’s Association (Institution Review Board number: 17/01/2025-9). As the study relied on anonymized clinical records, the requirement for informed consent was waived. All procedures complied with the principles of the Declaration of Helsinki and national data protection regulations.

## 3 Results

### 3.1 Demographic and clinical characteristics

Descriptive statistics for demographic variables and performance on the ACE-R by cognitive and mood diagnostic category are presented in Table 1.

**Table 1:** Participant demographics and ACE-R scores by cognitive status and mood disorder diagnosis.

Variable	MCI with mood disorder (*n* = 48)	MCI without mood disorder (*n* = 44)	Neurotypical with mood disorder (*n* = 44)	Neurotypical without mood disorder (*n* = 47)
Age	72.27 ± 5.80	70.16 ± 6.19	71.23 ± 6.85	69.45 ± 6.95
Sex	45 F/3 M	35 F/9 M	39 F/5 M	37 F/10 M
Education	11.85 ± 3.88	14.20 ± 3.62	14.02 ± 3.79	13.60 ± 3.85
ACE-R	80.81 ± 7.02	83.98 ± 6.56	91.55 ± 5.34	93.81 ± 4.34

Sex is reported as frequency (F: female, M: male). Other variables are reported as M ± SD. MCI, mild cognitive impairment; ACE-R, Addenbrooke’s Cognitive Examination–Revised.

Group comparisons between individuals with and without a mood disorder diagnosis indicated no significant differences between the two groups in terms of age, *t*(181) = 0.14, *p* = 0.891, Cohen’s *d* = 0.02, or education, (*t*(181) = −0.16, *p* = 0.876, Cohen’s *d* = −0.02). However, a significant difference was observed in sex distribution, (*χ*^2^(1) = 9.99, *p* = 0.002, Cramer’s *V* = 0.24). This effect was small to moderate in magnitude.

Means and standard deviations for the primary and secondary outcome measures across groups are reported in Table 2.

**Table 2:** Descriptive statistics for language measures and severity of depressive symptoms by cognitive status and mood diagnosis.

Cognition	Mood diagnosis	*n*	GDS	Fluency	Naming
MCI	With	48	7.63 ± 3.81	8.31 ± 2.22	8.27 ± 1.72
	Without	44	2.98 ± 2.80	8.91 ± 1.48	7.68 ± 1.67
	Total	92	5.44 ± 4.09	8.60 ± 1.92	7.99 ± 1.71
Neurotypical	With	44	6.59 ± 3.15	11.20 ± 1.23	9.59 ± 0.62
	Without	47	3.26 ± 2.58	11.83 ± 1.82	9.77 ± 0.48
	Total	91	4.90 ± 3.31	11.53 ± 1.59	9.68 ± 0.55
Total	With	92	7.14 ± 3.53	9.70 ± 2.32	8.90 ± 1.47
	Without	91	3.12 ± 2.67	10.42 ± 2.21	8.76 ± 1.59
	Total	183	5.18 ± 3.77	10.05 ± 2.29	8.83 ± 1.53

Values represent mean ± SD. MCI, Mild Cognitive Impairment; GDS, Geriatric Depression Scale.

### 3.2 Regression results

#### 3.2.1 Verbal fluency

For verbal fluency, model A with mood disorder diagnosis and cognitive status as predictors and age, education, and sex as covariates, was statistically significant (*F*(5, 177) = 35.63, *p* < 0.001, *R*^2^ = 0.50, adjusted *R*^2^ = 0.49). HC3 robust standard errors were employed to correct for heteroskedasticity in the residuals. Neurotypical participants performed at a higher level than those with MCI (*B* = 2.20, SE = 0.30, 95% CI [1.60, 2.79], *p* < 0.001) and participants without a mood disorder scored higher than those with a mood disorder (*B* = 0.62, SE = 0.25, 95% CI [0.12, 1.12], *p* = 0.015). With respect to demographic variables, older age predicted lower fluency (*B* = −0.06, SE = 0.02, 95% CI [−0.10, −0.02], *p* = 0.003) and higher education was associated with higher fluency (*B* = 0.15, SE = 0.04, 95% CI [0.08, 0.23], *p* < 0.001). In contrast, sex was not a significant predictor (*B* = 0.02, SE = 0.40, 95% CI [−0.78, 0.81], *p* = 0.965).

Model B, which additionally included depressive symptom severity, remained statistically significant (*F*(6, 163) = 28.16, *p* < 0.001, *R*^2^ = 0.51, adjusted *R*^2^ = 0.49). Cognitive status was the strongest predictor (*B* = 1.53, SE = 0.62, 95% CI [1.12, 1.94], *p* < 0.001) and education was positively associated with verbal fluency (*B* = 0.07, SE = 0.03, 95% CI [0.01, 0.14], *p* < 0.030). Age and sex were not significant predictors (*B* = −0.03, SE = 0.02, 95% CI [−0.07, 0.00], *p* = 0.086 and *B* = −0.51, SE = 0.35, 95% CI [−0.18, 1.20], *p* = 0.145, respectively), nor was a clinical diagnosis of a mood disorder (*B* = −0.26, SE = 0.24, 95% CI [−0.72, 0.21], *p* = 0.281). The coefficient for depressive symptom severity was not significant (*p* = 0.109, robust Wald test) and the model did not improve meaningfully over Model A (ΔAIC = −1.57). Thus, mood severity was not retained as a predictor.

To examine whether mood and cognition were differentially associated with phonemic and semantic fluency, separate multiple regression models were fitted for phonemic and semantic fluency using the Model A specification. Regression results for letter and category fluency are presented in Table 3.

**Table 3:** Regression analysis predicting letter and category fluency.

Predictor	Letter fluency		Category fluency	
	B (SE)	*p*	B (SE)	*p*
Cognitive status (Neurotypical—MCI)	2.93 (0.62)	<0.001	4.56 (0.68)	<0.001
Mood disorder diagnosis (without—with)	1.17 (0.53)	0.026	1.03 (0.58)	0.074
Age	−0.07 (0.04)	0.081	−0.16 (0.04)	<0.001
Education	0.33 (0.07)	<0.001	0.20 (0.07)	0.0068
Sex	0.23 (0.78)	0.765	0.17 (0.86)	0.844

Values represent unstandardized coefficients (B) with standard errors (SE) in parentheses.

The regression model for letter fluency was statistically significant, (*F*(5, 175) = 20.74, *p* < 0.001, *R*^2^ = 0.37, adjusted *R*^2^ = 0.35). Neurotypical participants outperformed those with MCI and participants without a mood disorder and those with higher levels of education demonstrated significantly better performance (see Table 3). Neither age nor sex were significant predictors.

The fitted model for category fluency was also significant (*F*(5, 175) = 25.41, *p* < 0.001, *R*^2^ = 0.42, adjusted *R*^2^ = 0.41). Cognitive status, education and age significantly contributed to the model. In contrast, mood disorder diagnosis did not reach statistical significance after covariate adjustment. Finaly, sex was not significantly associated with category fluency performance.

#### 3.2.2 Naming

For naming performance, Model A was statistically significant (*F*(5, 177) = 18.69, *p* < 0.001, *R*^2^ = 0.35, adjusted *R*^2^ = 0.33). As residual diagnostics indicated heteroskedasticity, HC3 robust standard errors were used to ensure more reliable estimates. With respect to predictors, cognitive status emerged as the only significant factor, with neurotypical participants scoring significantly higher than those with MCI, *B* = 1.46, SE = 0.21, 95% CI [1.05, 1.86], *p* < 0.001. Full regression coefficients and significance levels for the predictors are presented in Table 4.

**Table 4:** Regression analysis predicting naming performance (Model A).

Predictor	B (SE)	95% CI	*p*
Cognitive status (Neurotypical—MCI)	1.46 (0.21)	[1.05, 1.86]	<0.001
Mood disorder diagnosis (without—with)	−0.28 (0.19)	[−0.65, 0.09]	0.136
Age	−0.03 (0.02)	[−0.06, 0.00]	0.069
Education	0.05 (0.03)	[−0.02, 0.12]	0.132
Sex	0.43 (0.30)	[−0.17, 1.03]	0.155

Values represent unstandardized coefficients (B) with standard errors (SE) in parentheses and 95% confidence intervals (CI).

Model B extended Model A by adding GDS score as a predictor. The model remained statistically significant, (*F*(6, 163) = 15.55, *p* < 0.001, *R*^2^ = 0.36, adjusted *R*^2^ = 0.34) and the effect of cognitive status robust (*B* = 0.95, SE = 0.16, 95% CI [0,63, 1.27], *p* < 0.001). All other control variables failed to reach statistical significance, including mood disorder diagnosis (*B* = −0.20, SE = 0.18, 95% CI [−0.55, 0.15], *p* = 0.258), age (*B* = −0.02, SE = 0.0 1, 95% CI [−0.04, 0.00], *p* = 0.090), education (*B* = 0.04, SE = 0.03, 95% CI [−0.01, 0.09], *p* = 0.118) and sex (*B* = −0.25, SE = 0.22, 95% CI [−0.18, 0.68], *p* = 0.257). Mood severity was not significant (*p* = 0.384, robust Wald test) and model fit worsened (ΔAIC = +1.03). As a result, mood severity was once again not retained in the final model.

#### 3.2.3 Effect sizes for cognitive status

For both language measures, cognitive status was the strongest predictor. Neurotypical participants significantly outperformed those with MCI, with very large effect sizes (*d* = 1.64, 95% CI [1.32, 2.02] for fluency; *d* = 1.35, 95% CI [1.09, 1.66] for naming).

## 4 Discussion

The present study examined the predictors of language performance, focusing on verbal fluency and naming, using a two-model regression framework. Cognitive status emerged as the strongest predictor, with neurotypical participants demonstrating significantly better performance than those with MCI. In contrast, mood-related variables showed inconsistent effects. Diagnosis of mood disorder was a significant predictor of fluency but not naming, indicating that mood may preferentially affect effortful, executive aspects of word generation rather than basic lexical retrieval. Consistent with this interpretation, when phonemic and semantic fluency were analysed separately, mood disorder diagnosis was associated with letter fluency but not category fluency, whereas cognitive status remained robustly associated with both. Notably, depressive symptom severity did not independently predict performance in either outcome. Robust Wald tests of its coefficient were not significant, and inclusion of symptom severity did not improve model fit relative to simpler models. These findings indicate that while mood disorder diagnosis may be associated with fluency performance, severity of symptoms does not add explanatory value beyond diagnostic and demographic variables. This suggests that mood diagnoses may capture long-standing vulnerabilities relevant for language, whereas symptom severity at a given time may be less predictive.

Demographic factors also played a role, especially for fluency. Higher education was associated with better fluency performance, while older age predicted reduced fluency. These effects were evident for both phonemic and semantic fluency, with age showing a stronger association with category fluency. This pattern was not observed for naming, where neither age nor education reached statistical significance. Sex did not significantly predict performance in either outcome. Together, these results suggest that fluency, particularly its semantic component, may be more sensitive than naming to demographic influences.

The influence of demographic variables generally aligns with existing research. Educational attainment has been linked to improved verbal fluency performance (Cintoli et al., 2024; Karstens et al., 2024), supporting the cognitive reserve hypothesis (Stern et al., 2020). According to this hypothesis, higher education provides compensatory resources that protect against the effects of cognitive decline. In contrast to previous findings, however, education did not significantly influence naming performance in our sample. This may reflect the nature of the task and the limited number of the stimuli. In ACE-R, naming is assessed with confrontation naming of only 10 relatively familiar pictures, items that may be insensitive to educational background compared to more generative tasks such as verbal fluency. This interpretation is further supported by our finding that education was significantly associated with both phonemic and semantic fluency, indicating that educational experience benefits both executive search processes and semantic retrieval demands. Regarding age, its role in language performance is not uniform. The absence of age effects on naming in our study is in line with earlier findings that lexical retrieval may be relatively preserved compared to more complex generative tasks in the earlier stages of decline (Laws et al., 2010). Indeed, evidence from meta-analyses shows that confrontation naming accuracy remains stable until around age 70, after which age-related decline becomes more evident, particularly in individuals with lower educational attainment (Laws et al., 2010; Wen and Dong, 2023). This moderating role of education suggests that higher educational levels delay the onset of naming decline by nearly a decade. Even so, while naming accuracy may be maintained, response efficiency tends to diminish with advancing age, as reflected in slower responses and increased tip-of-the-tongue states (Hamberger et al., 2022). Finally, no significant sex effects were observed for fluency or naming. This is consistent with evidence from a recent meta-analysis indicating only a small female advantage in phonemic fluency, and negligible or inconsistent sex differences in semantic fluency, often varying by semantic category (Hirnstein et al., 2023).

The study’s findings confirm that language tasks, are highly sensitive to cognitive decline, and align with prior evidence that verbal fluency deficits are an early marker of cognitive impairment (McDonnell et al., 2020; Sharma and Malek-Ahmadi, 2023) and that MCI affects lexical access and semantic organization (Liampas et al., 2023; Seidman et al., 2025). The very large effect sizes observed here reinforce the view that verbal fluency is sensitive to early cognitive change as it relies on multiple cognitive processes, including executive control, strategic lexical search, and semantic organization. Consistent with this, cognitive status was robustly associated with both phonemic and semantic fluency, suggesting involvement of both executive retrieval and semantic network processes.

The role of mood was more nuanced. Mood disorder diagnosis was associated with fluency, but not naming, consistent with evidence that depression and other mood disorders disproportionately affect tasks requiring effortful lexical search and executive control (Fossati et al., 2003; Snyder, 2013). When fluency was decomposed, this association was specific to phonemic (letter) fluency and was not observed for semantic (category) fluency, suggesting that a mood disorder diagnosis in our sample was primarily linked to executive control and retrieval demands rather than disruption of semantic memory stores. Although earlier studies reported stronger depression-related effects on phonemic than semantic fluency, more recent evidence indicates that both components may be affected in depression, with semantic fluency often showing comparable or greater impairment (Kriesche et al., 2023; Henry and Crawford, 2005). In late-life and mild depression, cognitive effects are often subtle and task-dependent, with deficits emerging more clearly in higher-demand executive tasks while more automatic processes remain relatively preserved (Butters et al., 2004; Snyder, 2013). Given that our sample exhibited mild-to-moderate depressive symptoms, the selective effect on phonemic fluency is consistent with this pattern of executive inefficiency.

Importantly, the differential pattern between phonemic and semantic fluency helps to interpret the effects of cognitive status observed in the analyses. The study’s findings are consistent with evidence that semantic fluency is particularly sensitive to early neurocognitive decline in MCI (e.g., Olmos-Villaseñor et al., 2023), while also extending this work by showing that phonemic fluency is additionally influenced by mood disorder diagnosis. This dissociation suggests that semantic fluency primarily reflects disease-related disruption of semantic networks, whereas phonemic fluency captures combined effects of cognitive decline and mood-related executive dysfunction.

However, mood severity did not independently predict outcomes once cognition and demographic variables were considered. This contrasts with longitudinal findings showing that higher depressive symptom severity predicts poorer cognitive trajectories, including fluency performance (Dai et al., 2024), and that both the severity and duration of depression contribute to cognitive decline over time (Lu et al., 2025). Neither of these studies directly examined confrontation naming, but their results suggest that symptom severity may have cumulative effects on cognition that are less easily captured in cross-sectional analyses. The discrepancy may therefore reflect differences in study design, with longitudinal approaches being more sensitive to the cumulative effects of mood symptoms over time.

It must be noted that both MCI and mood disorders are linked to structural and functional alterations in brain regions that are critical for language, including the prefrontal cortex, hippocampus, and temporal lobes and are associated with impairments in verbal fluency and naming (Peelle, 2019; Trifu et al., 2024). In mood disorders, chronic inflammation, disrupted neurogenesis, and neurotransmitter imbalances (e.g., in serotonin and dopamine systems) are thought to underlie cognitive impairments. Depression, in particular, has been associated with reduced hippocampal neurogenesis due to chronic stress, thereby affecting cognitive processes (Hanson et al., 2011). Evidence indicates that hippocampal subfield volumes differ between cognitive decline and mood disorders, with atrophy in the left presubiculum observed in amnestic MCI/AD but not in mood disorders, suggesting a potential biomarker for distinguishing neurodegenerative from affective conditions (Hansen et al., 2021). In addition, patients with MCI often exhibit altered patterns of hippocampal activation during memory encoding, which are interpreted as compensatory neural responses recruited to counteract cognitive decline (Kircher et al., 2007). Thus, hippocampal dysfunction may compromise the memory-based processes that support lexical retrieval, semantic integration, and discourse-level functions, thereby contributing to language impairments such as verbal fluency and naming deficits (Covington and Duff, 2016; Duff and Brown-Schmidt, 2012). In line with this, the recently proposed L∪M (Language/Union/Memory) model conceptualizes language and memory as interacting systems within large-scale brain networks, providing a unifying framework for understanding how hippocampal dysfunction and network-level alterations translate into language–memory deficits in aging and neuropsychiatric disorders (Roger et al., 2022).

Several limitations should be acknowledged and considered when interpreting the validity and generalizability of the findings. First, because participants were recruited from a memory clinic, the findings are most applicable to similar clinical populations and may not generalize fully to community-based samples. Second, although the overall sample size was sufficient to detect large effects, subgroup sizes were modest when participants were stratified by both cognitive status and presence or absence of mood disorder, limiting the power to examine potential combined effects. Third, mood disorder classifications were based on ICD-10 codes assigned in routine clinical practice, based on reported history and clinical judgment, which may vary in accuracy or specificity. Potential diagnostic misclassification may have introduced measurement error and could have attenuated associations involving mood status. Fourth, language assessment was restricted to two measures, naming and verbal fluency. While these measures are widely used in clinical practice, they do not capture the full complexity of language functions, such as semantic processing or discourse. In addition, for the naming task, reaction-time data were not available in this retrospective dataset, and the small number of naming items may have limited sensitivity, reducing our ability to detect subtle group differences. Finally, several potentially influential variables (e.g., medication use, comorbidities, functional limitations, and lifestyle factors) were not available in a consistent or standardized form across participants and therefore could not be included as covariates, which may have influenced the observed associations.

Future research should address these limitations through longitudinal designs to clarify language decline trajectories in older adults with comorbid MCI and mood disorders. More comprehensive language batteries with more sensitive naming measures, along with detailed clinical, lifestyle, and biomedical data (e.g., medication use, comorbidities, neuroimaging), is needed to better capture the interplay between cognition, mood, and language.

## 5 Clinical implications

By employing a standardized neuropsychological battery within a primary healthcare context, the study provides an ecologically valid assessment of the interaction between mood, cognition, and language performance. This approach offers important insights for clinical practice, as understanding the association of mood disorders and cognitive decline with language performance can support more accurate differential diagnosis and inform the development of targeted intervention strategies for older adults at risk.

In terms of diagnostic differentiation, the study shows that language deficits in verbal fluency and naming are more strongly associated with cognitive decline than with mood disorders, highlighting the need for clinicians to avoid attributing word-finding problems to depression, as this may delay accurate identification of MCI or dementia. Importantly, mood disorder diagnosis was selectively associated with phonemic fluency, whereas semantic fluency and naming were primarily driven by cognitive status, suggesting that separating fluency components may improve the specificity of cognitive profiling in older adults with mood disorders or suspected neurocognitive decline. Regarding screening tools, the verbal fluency and naming tasks from the ACE-R can be employed as sensitive and cost-effective measures in primary care and memory clinics, facilitating early detection of cognitive decline.

Moreover, the fact that higher educational attainment was associated with better language performance underscores the importance of considering educational background in test interpretation and applying education-adjusted norms. Furthermore, it supports the cognitive reserve hypothesis, promoting lifelong learning as a possible protective factor against cognitive decline.

Managing mood disorders remains clinically relevant, as mood diagnosis was associated with reduced language performance even though symptom severity did not directly predict deficits. This pattern suggests that chronic mood conditions may exert persistent effects on cognitive-linguistic functioning and may influence participation and communication more broadly.

In conclusion, the present findings highlight the fact that while mood disorders may contribute to variability in language performance, cognitive decline is the dominant factor influencing verbal fluency and naming in older adults. Mood-related effects on fluency appeared selective, with stronger associations for phonemic than semantic fluency. The role of education as a resilience factor underscores the value of integrating demographic context into clinical assessments. These results reinforce the importance of early identification of cognitive impairment and support the use of language tasks as sensitive markers in routine assessment. Separating phonemic and semantic fluency may further enhance clinical specificity when evaluating older adults with mood disorders or suspected neurocognitive decline. Supporting communication and quality of life in older adults calls for coordinated assessment and intervention that address cognitive, emotional, and educational aspects.

## Data Availability

The data analyzed in this study is subject to the following licenses/restrictions: the data that support the findings of this study are available from the corresponding author upon reasonable request. Requests to access these datasets should be directed to Nomiki Karpathiou, n.karpathiou@go.uop.gr.
